# Mechanisms underlying the impact of environmental factors (physical, ecological, social) on infectious diseases

**DOI:** 10.1186/s42522-026-00234-3

**Published:** 2026-07-27

**Authors:** Mengjie Wang, Jun Wan, Qi Chen, Maolin Zhou, Xueyan Ma, Tengfei Liu, Quan Sun, Juan Liu, Junyi Ge, Tao Liu, Weishu Li, Yijing Tao, Shaobin Wang, Tiegang Liu, Bo Han, Chen Bai

**Affiliations:** 1 School of Traditional Chinese Medicine, Beijing University of Chinese Medicine, Beijing, 100029 China; 2 Shenzhen Hospital (Longgang), Beijing University of Chinese Medicine, Guangdong, 518172 China; 3 Guangdong Provincial Key Laboratory for Climate Change and Natural Disasters, Sun Yat-sen University, Guangdong, 510275 China; 4 School of Economics and Management, Open University of China, Beijing, 100039 China; 5 The RUC Leading Group for Talents, Renmin University of China, Beijing, 100872 China; 6 Institute of Immunization, Guangdong Provincial Center for Disease Control and Prevention, Guangdong, 511430 China

**Keywords:** Infectious diseases, Environmental factors, Climate change, Urbanization, Public health

## Abstract

The occurrence, transmission, and spread of infectious diseases are closely linked to environmental factors. As climate change intensifies, shifts in temperature, precipitation, and extreme weather events are modifying pathogen survival, vector ecology, and the geographical range of infectious diseases. At the same time, ecosystem degradation, land-use change, urbanization, poverty, and social unrest further shape population exposure, healthcare access, and disease vulnerability. This narrative review synthesizes the mechanisms by which physical, ecological, and social environmental factors influence infectious disease transmission. Unlike reviews that focus on single determinants, this review adopts an integrated framework to examine both individual mechanisms and cross-domain interactions. Guided by the One Health framework, we link these mechanisms to the human-animal-environment interface and highlight how environmental change can promote pathogen spillover, amplification, and spread. By clarifying these pathways, this review provides a theoretical basis for integrated infectious disease prevention, public health policy, and future research.

## Clinical trial number

Not applicable.

Seasonal influenza epidemics, the drug-resistant tuberculosis crisis, and the global public health emergency caused by COVID-19 demonstrate the continuing threat posed by emerging and re-emerging infectious diseases [1, 2]. Vaccines remain essential for preventing infection, reducing transmission, and lowering epidemic risk. However, outbreaks can still occur even when vaccine coverage is relatively high. Global infectious disease burden data (Table 1) indicate that infectious diseases, including vaccine-preventable diseases, are unevenly distributed across regions. This uneven distribution suggests that infectious disease risk is shaped not only by biomedical interventions but also by broader environmental conditions.

In this review, environmental factors are defined broadly. They include physical factors, such as climate change; ecological factors, such as ecosystem alteration and biodiversity loss; and social factors, such as urbanization, infrastructure, health education, poverty, and armed conflict. This definition differs from a narrow focus on physical exposure alone. It reflects the fact that infectious disease transmission is shaped by interactions among climate, ecosystems, human behavior, infrastructure, and health systems. Understanding infectious disease epidemiology therefore requires a multidimensional framework that captures these interacting determinants.

**Table 1:** Global infectious disease data, 2023

Type	Diseases	African Region	Region of the Americas	South-East Asia Region	European Region	Eastern Mediterranean Region	Western Pacific Region	Global
SDG3 Diseases	New HIV infections (per 1000 uninfected population)	0.55	0.16	<0.1	0.18	<0.1	<0.1	0.17
	Tuberculosis incidence (per 100 000 population)	206	33	234	24	116	97	134
	Malaria incidence (per 1000 population at risk)	226.85	3.55	2.32	0	17.88	2.27	60.44
	Hepatitis B surface antigen (HBsAg) prevalence among children under 5 years (%)	2.5	0.1	0.4	0.3	0.8	0.3	0.9
	Reported number of people requiring interventions against NTDs	518 582 990	30 727 196	787 674 482	5 890 441	77 975 579	74 042 993	1 494 893 681
Immunization Diseases	Congenital Rubella Syndrome (per 10,000 live births)	0.1	0	0.1	0	0.6	0.4	0.2
	Diphtheria (per 1,000,000 total population)	23.9	0	2.2	0.3	0.7	2.1	4.2
	Invasive meningococcal disease (per 1,000,000 total population)	2.6	1.6	0.4	4.1	0.4	0.3	1.9
	Japanese Encephalitis	0	-	0.9	-	0	0.3	0.5
	Measles (per 1,000,000 total population)	343	0.1	37	65.3	122.8	11.4	85.5
	Mumps (per 1,000,000 total population)	761.5	18.8	18.7	11.3	27.1	64.1	95.2
	Pertussis (per 1,000,000 total population)	8.4	6.9	5.6	95.1	5.5	23.8	22.7
	Poliomyelitis (per 1,000,000 < 15 population)	1.4	0	-	0.4	0.2	0.1	0.7
	Rubella (per 1,000,000 total population)	21.9	0	1.8	0.3	6	0.7	4.6
	Tetanus (per 1,000,000 total population)	13.2	0.5	0.1	0.1	1	6.5	3.6
	Typhoid (per 1,000,000 total population)	5743.2	3.6	571.2	1.5	41.4	75.8	1044.7
	Yellow Fever (per 1,000,000 total population)	0.4	0.1	0	0	0	0	0.2

Note: SDG3, Sustainable Development Goal 3 (good health and well-being).Data sources: WHO Global Health Observatory (https://www.who.int/data/gho/whs-annex/) and WHO Immunization Data Portal (https://immunizationdata.who.int/global?topic=&location=) (accessed 2 December 2025)

**Fig. 1:**
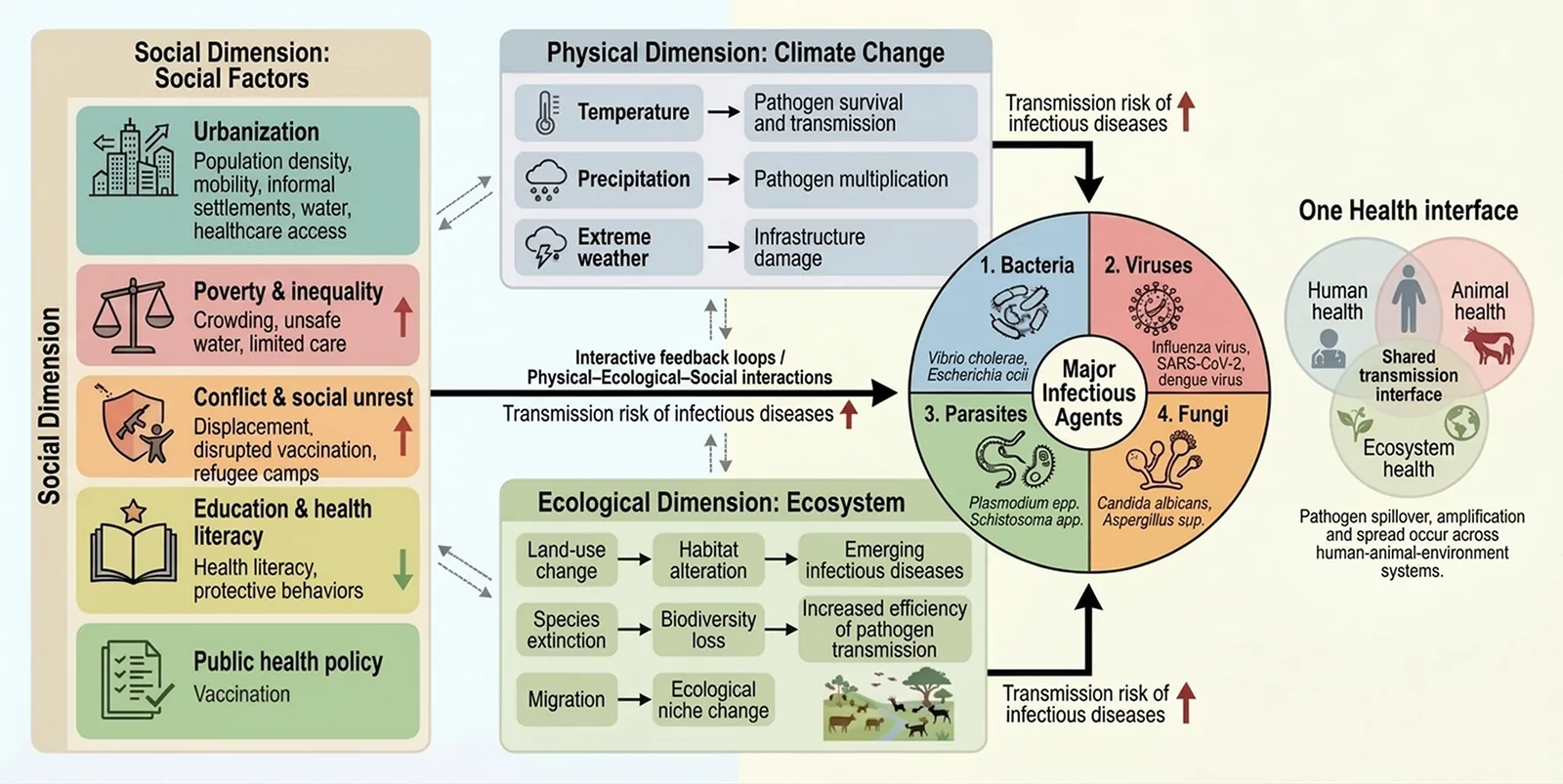
Multidimensional mechanisms linking environmental change to infectious disease transmission under the One Health framework. The transmission risk of infectious diseases is shaped by interactions among physical, ecological, and social dimensions. Physical factors associated with climate change, including temperature change, altered precipitation, and extreme weather events, influence pathogen survival, pathogen multiplication, transmission potential, and infrastructure stability. Ecological factors, including land-use change, habitat alteration, species extinction, biodiversity loss, migration, and ecological niche change, can promote the emergence of infectious diseases and increase pathogen transmission efficiency. Social factors, including urbanization, poverty and inequality, conflict and social unrest, education and health literacy, and public health policy, affect transmission risk by influencing population density, mobility, informal settlements, water and healthcare access, crowding, vaccination, and protective behaviors. Through physical-ecological-social feedback loops, these factors collectively increase the transmission risk of major infectious agents, including bacteria, viruses, parasites, and fungi. Under the One Health framework, pathogen spillover, amplification, and spread occur across the shared human-animal-environment interface

Environmental drivers influence infectious disease transmission through several pathways. Climate change can alter temperature and precipitation patterns, thereby affecting pathogen survival, vector distribution, and host exposure [3–5] (Fig. 1). Ecosystem degradation and biodiversity loss can reshape reservoir host communities and modify the probability of pathogen spillover [6, 7]. Social conditions, including population density, mobility, sanitation, healthcare access, and health literacy, determine whether environmental hazards translate into sustained transmission. For example, temperature and humidity can influence influenza virus transmission [8, 9], while interactions between pollution and socioeconomic disadvantage may contribute to high infectious disease burdens in vulnerable regions [10].

Based on the One Health framework, this narrative review synthesizes the mechanisms through which physical, ecological, and social environmental factors influence infectious disease transmission. Unlike previous reviews that often focus on a single category of determinants, this review examines these dimensions together and emphasizes their interactions. We first discuss climate-related mechanisms, including temperature variation, precipitation change, and extreme weather events. We then review ecosystem-related mechanisms, including biodiversity loss, land-use change, and ecological niche shifts. Next, we examine social determinants, including urbanization, poverty, conflict, education, and policy. Finally, we analyze cross-domain interactions and discuss future research directions. Through this structure, the review aims to clarify how environmental change influences infectious disease risk at the human-animal-environment interface.

This narrative review draws on literature identified through PubMed, Web of Science, and Google Scholar searches for publications up to June 2026. Search terms included “environmental factors,” “physical environment,” “ecological factors,” “social factors,” “climate and health,” and related terms. We also screened the reference lists of key reviews and empirical studies. Because this is a narrative review, we did not apply formal quantitative inclusion criteria or quality assessment tools. Instead, we prioritized studies that provided empirical or mechanistic evidence relevant to infectious disease transmission across physical, ecological, or social dimensions.

## Climate change and infectious diseases: climate sensitivity and multidimensional drivers

Climate change influences infectious disease transmission by altering pathogen survival, vector distribution, reservoir activity, and human exposure patterns. It affects vector-borne, respiratory, and waterborne diseases through changes in temperature, precipitation, and extreme weather events. Evidence suggests that 58% of known human infectious diseases are climate-sensitive, and more than 200 diseases, including dengue fever, malaria, and anthrax, have been aggravated by climate-related hazards [11].

### Temperature variation: multi-level mechanisms involving pathogens, vectors, and hosts

Temperature affects infectious disease transmission by influencing pathogens, vectors, and hosts. It shapes pathogen survival outside the host, vector breeding and competence, and human behaviors that determine exposure. Therefore, temperature is not only a climatic variable but also a mechanistic driver that links environmental change to biological susceptibility and population-level transmission risk.

At the pathogen and vector level, temperature influences disease transmission through three main pathways. First, warm and humid conditions favor mosquito breeding, shorten mosquito life cycles, increase vector abundance, and expand the geographical suitability of mosquito-borne diseases [12–16]. Compared with 1951–1960, the suitability of *Aedes albopictus* and Aedes aegypti for dengue virus transmission increased by approximately 27–28% during 2013–2022. Warming also extended the seasonal transmission window by 13–14% for these two species [17]. Second, temperature affects viral stability outside the host. Low temperatures can increase influenza virus transmission and prolong host infectiousness. In animal experiments, infected guinea pigs housed at 5 °C shed virus for approximately 40 h longer than those housed at 20 °C [18, 19]. A nonlinear association has also been observed between atmospheric temperature, absolute humidity, and the effective reproductive number of SARS-CoV-2, with transmission peaking at 10.2 °C and 6.6 g/m³ absolute humidity [20]. Third, temperature can influence virus-vector compatibility. West Nile virus transmission often peaks between 23 °C and 26 °C [21], while dengue transmission probability may reach 60% when daily mean temperature is approximately 20 °C with substantial diurnal temperature variation [22].

At the host level, temperature affects disease risk by changing both exposure and immune defense. Cold weather encourages indoor crowding and reduces ventilation, increasing the risk of person-to-person transmission [23, 24]. Low temperatures can also impair innate immune function in the respiratory mucosa, thereby increasing susceptibility to influenza virus [25]. The risk of influenza incidence has been reported to peak at 5.1 °C [26]. Experimental evidence suggests that short-term exposure to low temperatures can inhibit immune cells such as macrophages, reducing respiratory pathogen clearance [27]. High-temperature stress can also affect host physiology by damaging intestinal barrier integrity, increasing intestinal permeability, and promoting systemic inflammation [28, 29]. The risk of bacillary dysentery has been reported to peak at 29 °C [30]. For vector-borne diseases such as malaria, temperature may further influence host inflammatory responses and disease progression [31]. When mean temperature exceeds 10 °C, each 5 °C increase is associated with a 22% rise in malaria cases [32].

### Changes in precipitation patterns: bidirectional effects on vector breeding and waterborne transmission

Changes in precipitation affect infectious disease transmission by altering surface hydrology, vector habitats, and access to safe water [33]. Excessive rainfall and insufficient rainfall can both increase disease risk, but through different mechanisms.

Excessive precipitation can create standing water and expand breeding sites for mosquitoes and other vectors [34–37]. This may increase vector density and enhance pathogen transmission efficiency [38]. By contrast, insufficient precipitation can increase risk through water scarcity. Households may store water more frequently, creating artificial breeding sites for mosquitoes and increasing exposure to infected vectors [39]. Water scarcity may also force communities to rely on unsafe water sources [40]. In addition, drought conditions can increase contact among humans, livestock, wildlife, rodents, and vectors, thereby increasing infectious disease risk [41].

These pathways do not cancel each other out. Excessive and insufficient precipitation usually occur in different seasons, locations, and ecological contexts. Their effects also depend on vector species, water-use behavior, sanitation conditions, and local infrastructure [42–44].

### Extreme weather events: outbreak mechanisms including biological damage

Climate change has increased the frequency and intensity of extreme weather events. Floods, droughts, and heatwaves are increasingly associated with infectious disease outbreaks [45, 46]. These events influence disease risk through three interconnected mechanisms: direct biological effects, disruption of infrastructure and health systems, and disaster-induced population displacement.

First, extreme weather can directly affect host susceptibility and pathogen survival. Extreme temperatures induce heat stress [47], exacerbate inflammatory responses, impair immune function, and weaken population-level immune responsiveness [48]. These changes increase susceptibility to infectious diseases. High temperatures can also alter the survival and replication of pathogens in the environment. For example, *Vibrio cholerae* grows rapidly in warm water bodies, increasing the risk of waterborne disease [49].

Second, extreme weather can damage infrastructure and health systems. Floods may destroy latrines, contaminate drinking water sources, damage housing, and create standing water that supports mosquito breeding. These changes increase the risk of enteric and mosquito-borne diseases [50–52]. Extreme events can also disrupt transportation, electricity, and medical supply chains, reducing healthcare access and weakening emergency response capacity [53, 54]. Drought-related water scarcity further compromises hygiene and healthcare services [55–57]. These mechanisms help explain the observed associations between extreme weather and dengue outbreaks [58], as well as the effects of China’s 2022 climate anomalies on infectious disease epidemics [59, 60].

Third, extreme weather can displace populations and accelerate cross-regional pathogen spread. Displaced populations may carry pathogens during incubation or active infection into receiving areas, creating importation risk [61]. Overcrowded shelters with limited sanitation create aggregation risk [51, 62]. At the same time, disruption of routine healthcare services may weaken population immunity and increase susceptibility to vaccine-preventable diseases [63].

## Ecosystem changes and their association with infectious diseases: core drivers and mechanistic overview

Ecosystem disturbance is an important driver of infectious disease emergence and transmission. Biodiversity loss, land-use change, and ecological niche shifts can alter reservoir host communities, vector habitats, and human-animal contact patterns. These ecological changes influence infectious disease risk by reshaping where pathogens persist, how they circulate among hosts, and when they cross into human populations.

### Biodiversity loss: dilution effect, amplification debate, and host community restructuring

Biodiversity loss can affect infectious disease risk by restructuring host communities. When species richness declines, competent reservoir hosts may represent a larger share of the remaining community. This can increase pathogen transmission among hosts and raise spillover risk [64]. Host community homogenization may also increase contact between humans and reservoir animals, making it easier for wildlife pathogens to cross species barriers [65, 66]. Predator loss can further alter transmission through trophic cascades. For example, reductions in large herbivores may change vegetation structure and increase the abundance of vectors such as mosquitoes [67].

However, the relationship between biodiversity and disease risk is not universal. Ecologists have long debated whether biodiversity consistently dilutes infectious disease risk or, under some conditions, amplifies it. This debate is well illustrated by Lyme disease. Ogden and Tsao [68] noted that high species diversity may reduce transmission by lowering the relative abundance of highly competent reservoir hosts such as the white-footed mouse. In other contexts, however, biodiversity loss may amplify disease risk by increasing the density of competent hosts or by altering community composition in ways that favor pathogen maintenance. Thus, the effect of biodiversity depends on reservoir host composition, pathogen ecology, and local environmental conditions.

### Land-use change: effects of habitat compression-population aggregation -contact amplification

Land-use change reshapes infectious disease transmission by altering ecological boundaries and contact patterns. Urbanization, agricultural intensification, and deforestation convert natural habitats into human-dominated landscapes [69]. These changes affect species distributions, interspecific contact rates, and pathogen transmission networks. Studies indicate that a 1% increase in urban land is associated with a 0.081% increase in infectious disease incidence and a 0.07% increase in mortality [70].

A key mechanism is the cascade of habitat compression, host aggregation, and contact amplification. Agricultural expansion, land consolidation, and deforestation fragment natural habitats and reduce native ecological niches. As a result, wildlife may be forced into residual habitat patches or human-dominated landscapes [71–73]. A 43-year ecological monitoring study in Central China supports this mechanism. Extensive land consolidation caused the local extinction of eight native rodent species, while striped field mice proliferated in human settlements [74]. Compressed habitats can increase reservoir host density, accelerate pathogen circulation among wild animals, and increase human exposure to infected wildlife [75, 76]. The loss of intact ecosystems also weakens ecological buffering functions, thereby increasing the risk of wildlife-borne pathogen spillover.

### Niche shifts: emerging infectious diseases driven by niche displacement and species invasion

An ecological niche refers to the range of resources, habitats, and interactions that allow a species to persist within an ecosystem. When climate change, land-use change, or biological invasion alters these conditions, species may shift their distributions and form new ecological relationships. These niche shifts can create new host-pathogen-vector interactions.

Poleward shifts in suitable habitats can expand the range of vector-borne diseases. For example, Ixodes ticks, the vectors of Lyme disease, have expanded northward and established stable endemic areas in Canada [77, 78]. Invasive species can also create new transmission networks. The longhorned tick (Haemaphysalis longicornis), native to East and Central Asia, has expanded into new regions and can carry multiple pathogens [79–82]. Such niche reconstruction can generate novel host-pathogen interactions and may contribute to the emergence of infectious diseases.

## Impact of social factors on infectious diseases

Social factors shape infectious disease transmission by influencing exposure, behavior, healthcare access, and the capacity of communities to respond to environmental risks. Urbanization, poverty, education, public health policy, and social unrest determine how physical and ecological hazards are translated into population-level disease burdens.

### Urbanization: population aggregation, mobility networks, and infrastructure imbalance

Urbanization influences infectious disease transmission through population density, mobility, and infrastructure. High population density increases the frequency of interpersonal contact and strengthens the connectivity of contact networks, thereby facilitating pathogen transmission [83, 84]. High-frequency urban social activities further expand the size and heterogeneity of contact networks, increasing the spread of respiratory pathogens [85]. Public transportation and cross-regional mobility create spatial diffusion chains of “mobility-contact-transmission” [86, 87]. Urban mobility may also increase zoonotic outbreak risk through trade and animal-related networks [88].

Infrastructure can either reduce or amplify infectious disease risk. Reliable water supply and sanitation systems reduce the transmission of waterborne diseases. Improvements in water supply infrastructure are negatively associated with cholera incidence, and better water supply continuity can reduce incidence to 0.81 [89]. Adequate healthcare resources also shorten delays in care-seeking and improve isolation and treatment capacity. However, rapid urbanization often produces uneven infrastructure development. In slums and informal settlements, poor sanitation promotes pathogen accumulation, while limited healthcare access delays case detection and prolongs transmission [90, 91].

These risks are not limited to humans. Poor water and sanitation systems can create shared exposure environments for people, livestock, and urban wildlife. A One Health study in Ongata Rongai, a rapidly growing peri-urban area in Nairobi, Kenya, illustrates this problem [92]. Rapid population growth and unplanned construction have increased overcrowding and competition for water. Rivers polluted by solid waste and sewage threaten both human and animal health. Damaged riparian ecosystems further intensify water shortages, health risks, and livelihood insecurity.

### Socioeconomic status: poverty-driven risks of infectious diseases

Poverty increases infectious disease risk through a cascade of exposure, susceptibility, and limited healthcare access. Economic hardship often forces disadvantaged groups to live in overcrowded housing with inadequate water and sanitation. This helps explain why infectious disease prevalence is higher in low-income settlements than in wealthier neighborhoods [93–96]. These living conditions increase daily exposure to polluted water, unsanitary surroundings, and poorly ventilated dwellings.

Unsafe water and inadequate sanitation facilitate fecal-oral transmission of enteric pathogens [97–99]. When residents rely on unregulated water sources, the risk of ingesting waterborne pathogens increases. Globally, approximately 1.7 billion cases of diarrhea occur each year, and about 90% of diarrheal deaths are attributable to poor sanitation, inadequate sanitary facilities, and unsafe water [100]. Poverty also delays healthcare-seeking and reduces access to diagnosis, isolation, and treatment, weakening the interruption of transmission chains [101].

The COVID-19 pandemic highlighted this cascade. Residents of Iranian slums struggled to implement protective measures because of limited space and poor sanitation, while unemployment and income loss further increased health risks [102]. Low-income households also relied more heavily on public transportation, increasing exposure risk [103].

### Social unrest: infectious disease crises induced by armed conflict

Armed conflict exacerbates infectious disease transmission by damaging sanitation infrastructure, disrupting immunization, weakening health systems, and forcing population displacement. Unlike climate-related disasters, conflict generates disease risk mainly through institutional breakdown and prolonged social instability.

Conflict damages water supply and sanitation systems, creating conditions for outbreaks. In the Gaza Strip, ongoing conflict has rendered 97% of groundwater unfit for drinking and has contributed to more than 200,000 cases of diarrhea [104]. In Afghanistan, prolonged conflict has been associated with high incidence rates of tuberculosis, malaria, and COVID-19. A fragile healthcare system and unequal resource distribution have further intensified this burden [105]. By destroying environmental and sanitary buffers, armed conflict increases human exposure to contaminated surroundings.

Conflict also disrupts immunization services. Damage to cold-chain systems and security threats to vaccination personnel can sharply reduce vaccine coverage, with reductions exceeding 20% points in high-intensity conflict areas [106]. The polio outbreak in Syria illustrates this mechanism: conflict reduced vaccination coverage from more than 80% to less than 50% [107]. Northern Syria also experienced its largest measles outbreak since elimination in 1999, with more than 30,000 clinically suspected cases reported in 2017 and 2018 [108].

Forced displacement further amplifies infectious disease risk. The conflict in Sudan has worsened disease transmission among displaced populations, and cross-border movement can introduce pathogens into neighboring countries [109]. Crowded refugee camps create favorable conditions for respiratory, contact-transmissible, and waterborne diseases. ProMED analyses indicate that measles, cholera, and cutaneous leishmaniasis are particularly prominent in camp settings [110]. Migration can also introduce pathogens into low-prevalence areas, causing imported outbreaks. For respiratory diseases such as COVID-19 and influenza, human mobility is strongly associated with transmission risk [111].

In short, armed conflict transforms social instability into infectious disease risk by simultaneously damaging environmental safety, vaccination systems, healthcare capacity, and population stability.

### Educational attainment and health literacy: knowledge-driven mechanisms of behavior internalization

Education and health literacy influence infectious disease risk because many preventive behaviors depend on knowledge, risk perception, and trust in health information. Educational attainment supports protection through three steps: knowledge acquisition, cognitive transformation, and behavioral internalization. Higher educational attainment improves understanding of transmission routes and prevention strategies. It also helps individuals translate health knowledge into protective behavior and identify false health information [112–114].

Empirical evidence supports this pathway. Health education can reduce the spread of tropical diseases and influenza by improving public awareness of transmission and prevention [115, 116]. Conversely, insufficient health knowledge can delay medical care. Low awareness contributes to the persistence of neglected tropical diseases among low-income populations [117].

### Public health policy: demand-oriented resource allocation and community engagement

Public health policy reduces infectious disease risk by identifying health needs, allocating resources, and guiding population behavior. Effective policies first recognize differences in risk and access across socioeconomic groups. They then reduce barriers to healthcare through targeted interventions, such as free vaccination services for low-income residents [118, 119].

Community engagement is also essential. Participation-based systems can increase public trust, improve compliance with preventive measures, and support the development of herd immunity [120]. Public health policy therefore acts as a coordinating mechanism. It links resources, infrastructure, behavioral guidance, and community participation to reduce transmission across populations and shared environments.

## Mechanisms of the impact of environmental factor interactions on infectious diseases

The preceding sections examined physical, ecological, and social determinants separately. In practice, these factors rarely act alone. Climate hazards, ecosystem change, and social vulnerability often converge in the same places and populations. This section therefore focuses on cross-domain interactions and explains how combined environmental pressures shape pathogen survival, vector and reservoir dynamics, human exposure, and outbreak amplification.

### Physical-ecological interactions: synergistic drivers of climate and ecology

Climate change affects ecosystems by altering temperature and precipitation regimes. These changes influence resource availability, energy flow, species interactions, dispersal, and seasonal phenology [121]. Warming can also reduce food-web resilience and contribute to local species extirpation [122–125]. These ecological changes, in turn, influence vector survival, distribution, biting activity, and pathogen replication within vectors [126–129]. Thus, the epidemiological effects of climate change depend not only on direct effects on pathogens and vectors but also on how ecosystems reorganize under climatic stress.

Deforestation illustrates this interaction. By altering local microclimates, deforestation can increase local temperatures and shorten the development time of Plasmodium parasites within Anopheles mosquitoes, thereby increasing malaria transmission risk [130]. In the Amazon, deforestation can also promote infectious disease spread by changing climatic conditions and increasing opportunities for human-wildlife contact [66]. In these cases, climate becomes epidemiologically important because ecological change reshapes where humans, wildlife, and vectors overlap.

Climate-ecology interactions can also increase zoonotic disease risk by compressing wildlife habitats and changing species movement. Resource stress may force animals into agricultural or urban landscapes, increasing contact among wildlife, livestock, vectors, and humans. El Niño events provide one example: changes in food resource distribution can induce fruit bats to move into agricultural-urban systems, facilitating *Hendra virus* transmission [131]. In the Western Ghats of India, the interaction between low mammal species abundance and deforestation increases the risk of Kyasanur Forest disease outbreaks [132]. These examples show that climate does not operate independently of ecology. Instead, climate-induced ecological change can restructure the human-animal-environment interface.

### Physical-social interactions: climate hazards, social vulnerability, and exposure amplification

Climate hazards do not affect all populations equally. Their health impacts are shaped by social vulnerability, including poverty, inadequate housing, insufficient infrastructure, limited healthcare access, poor nutrition, and constrained mobility. These social disadvantages can amplify the health risks associated with climate change by increasing exposure, weakening host resilience, and limiting timely response.

This interaction operates through three pathways. First, climate-related livelihood and food-system stress can increase biological susceptibility. Food shortages and rising food prices may worsen malnutrition, weaken immune function, and increase susceptibility to and severity of diseases such as tuberculosis [133]. Second, climate-induced displacement can concentrate vulnerable populations in receiving areas where overcrowding, unsafe water, poor sanitation, and limited medical resources facilitate transmission [134]. Third, behavioral adaptation to climate stress can reshape contact patterns. For example, low temperatures encourage indoor gathering and reduced ventilation, which can facilitate respiratory virus transmission [135].

Infrastructure is a key mediator between climate hazards and infectious disease outcomes. Climate-related environmental triggers interact with socioeconomic vulnerabilities, such as poor living conditions and limited medical care, to increase susceptibility and transmission risk [136–138]. Floods provide a clear example. In regions with lower economic development and inadequate infrastructure, flood exposure significantly increases hospitalizations for foodborne and waterborne diseases, and this elevated risk can last up to 26 weeks [139]. In urban informal settlements in India and Indonesia, poor sanitation, limited healthcare services, and frequent flooding jointly contribute to high rates of respiratory, gastrointestinal, and vector-borne diseases [140].

Therefore, climate impacts are not determined by physical exposure alone. They are filtered through social conditions that determine who is exposed, how intense the exposure is, and whether transmission can be interrupted.

### Ecological-social interactions: human-modified environments and pathogen exposure

Ecological-social interactions occur when human activities reshape ecological conditions, and these altered conditions then modify pathogen exposure. Land use, economic activity, settlement patterns, and pollution can all restructure the environments in which humans, animals, vectors, and pathogens interact.

Human-modified environments can create new opportunities for pathogen transmission. Derelict infrastructure, fragmented green space, stagnant water, and uncontrolled vegetation may increase the abundance of commensal pests and vectors [141]. A study in a temporary settlement in Kathmandu, Nepal, illustrates this risk. Viral surveillance of small mammals detected multiple potential pathogens, and nearly half of residents reported direct contact with rodents and shrews [142]. This finding highlights the vulnerability of urban-edge settlements, where social marginalization and altered ecological conditions overlap.

Air pollution represents another socio-ecological exposure pathway. Fine particulate matter (PM2.5), generated largely by industrial emissions and vehicle exhaust, is a major environmental health risk [143]. PM2.5 can damage the respiratory mucosal barrier and impair immune function, increasing susceptibility to viral respiratory infections [136]. Quantitative studies indicate that each 10 µg/m³ increase in PM2.5 exposure is associated with an approximate 5–8% increase in respiratory infectious disease incidence [144].

### Integrated physical-ecological-social interactions: multidimensional synergy and a one health perspective

Physical, ecological, and social factors often converge to produce infectious disease risk. Climate change, deforestation, and urbanization jointly increase zoonotic disease risk by reshaping ecological and geographical boundaries. Climate and land-use change shift the distribution of vectors and reservoir hosts, while urbanization increases human density and contact opportunities. Together, these processes facilitate both pathogen spillover and post-spillover transmission [145–148].

Climate-related displacement further illustrates this multidimensional synergy. Weather-related disasters displaced approximately 32 million people in 2022, representing a 41% increase compared with 2008 [149]. Temperature anomalies, irregular precipitation, and extreme weather can alter pathogen habitats and environmental survival conditions [150]. At the same time, displaced communities often face overcrowding, inadequate sanitation, limited medical resources, and insecure living conditions. These social vulnerabilities magnify infection risk and accelerate outbreak spread [151].

From a One Health perspective, infectious disease transmission is a cross-system process occurring at the human-animal-environment interface. Physical factors affect pathogen survival and vector distribution. Ecological factors alter reservoir hosts, species interactions, and habitat structure. Social factors shape exposure, mobility, healthcare access, and response capacity. These dimensions interact through feedback loops that drive pathogen spillover, amplification, and spread. Single-domain interventions are therefore unlikely to fully interrupt transmission chains. Integrated governance across human, animal, and ecosystem health is needed to reduce infectious disease risk.

## Limitations and future research directions

This narrative review synthesizes existing evidence on how physical, ecological, and social environmental factors influence infectious disease transmission. Although the literature provides substantial mechanistic insight, several limitations should be acknowledged.

First, this review is not a systematic review. We did not use a fully reproducible search, screening, or quality assessment protocol. This approach allowed broad synthesis across domains, but it also introduces the possibility of selective citation and author bias. Future studies should conduct systematic reviews and meta-analyses to provide more reproducible and quantitative evidence.

Second, much of the available evidence comes from observational epidemiological studies. These studies can identify associations between environmental factors and infectious disease risk, but they often cannot establish causal pathways or quantify the relative contribution of each determinant. Future research should apply stronger causal inference methods and explicitly model interaction terms, nonlinear relationships, and feedback mechanisms.

Third, the global distribution of evidence is uneven. Many mechanisms are better documented in regions with stronger surveillance systems, while data remain limited in low-resource and ecologically diverse settings. Future research should strengthen environmental and epidemiological surveillance in data-scarce regions. This would improve the generalizability of mechanistic conclusions across different socioeconomic and ecological contexts.

Fourth, pathogen evolution and changing host immunity may alter the relationship between environmental factors and disease transmission. Prior infections, vaccination, and pathogen adaptation can reshape population-level susceptibility. As a result, some mechanisms summarized in this review, such as temperature-dependent transmission, may change over time. Future research should develop dynamic models that integrate environment, pathogen evolution, and host immunity.

## Conclusion

This narrative review has summarized the mechanisms through which physical, ecological, and social environmental factors influence the emergence and transmission of infectious diseases. Climate change affects pathogen survival, vector ecology, host susceptibility, and extreme weather-related outbreaks. Ecosystem changes, including biodiversity loss, land-use change, and niche shifts, reshape reservoir host communities and pathogen spillover risk. Social factors, including urbanization, poverty, conflict, education, and public health policy, influence exposure, behavior, healthcare access, and response capacity.

The impact of environmental factors on infectious diseases is therefore complex and multidimensional. These factors rarely act in isolation. Instead, they interact at the human-animal-environment interface, where physical hazards, ecological disruption, and social vulnerability can reinforce one another. The One Health framework provides a useful approach for understanding these interactions and designing integrated prevention strategies. Effective infectious disease control requires coordinated action across climate adaptation, ecosystem protection, infrastructure improvement, health education, surveillance, vaccination, and equitable healthcare access.

## Data Availability

Not applicable.
